# Assessing Quality of Action Research Using the Quality Assessment Action Research Checklist (QuARC): A Hybrid Systematic Narrative Review

**DOI:** 10.1111/jan.70607

**Published:** 2026-04-20

**Authors:** Mary Casey, David Coghlan, Áine Carroll, Tom Buckley, Diarmuid Stokes

**Affiliations:** ^1^ UCD School of Nursing, Midwifery & Health Systems University College Dublin Dublin 4 Ireland; ^2^ Trinity Business School University of Dublin, Trinity College Dublin 2 Ireland; ^3^ School of Medicine University College Dublin Dublin 4 Ireland; ^4^ National Rehabilitation Hospital Dun Laoghaire Co Dublin Ireland; ^5^ Susan Wakil School of Nursing and Midwifery, Faculty of Medicine and Health The University of Sydney Camperdown New South Wales Australia; ^6^ The Library University College Dublin Dublin 4 Ireland

## Abstract

**Aim:**

To evaluate the quality of action research studies using the Quality Assessment Action Research Checklist (QuARC) and to assess its utility as a tool for quality appraisal.

**Design:**

A hybrid systematic narrative review following Turnbull et al.'s six‐stage methodology and reported in accordance with PRISMA 2020 guidance.

**Data Sources:**

Scopus was searched for author self‐identified action research studies published between January 2020 and March 2024.

**Review Methods:**

Two reviewers independently selected studies meeting inclusion criteria: health science action research papers addressing any or all of QuARC's four quality factors. A scoring system was used to capture each of QuARC's 17 quality items, which was scored as 0 (absent), 0.5 (partial) or 1 (comprehensive). Narrative synthesis was undertaken across the four QuARC domains.

**Results:**

Thirty‐two studies met the inclusion criteria. Reporting frequencies across QuARC were: Context (92.5%, mean = 3.7/4), Quality of Relationships (55% mean = 2.2/4), Quality of Action Research Process (62.5% mean = 2.5/4), and Quality of Outcomes (62.5% mean = 3.1/5). Reporting gaps were most evident in reflexive co‐analysis, relational evaluation and explicit theoretical contribution.

**Conclusion:**

Global reporting of rigour and quality in action research remains inconsistent. QuARC functioned both as an appraisal instrument and as an analytic lens, revealing systematic patterns in how action research privileges practical change over theoretical articulation and reflexive relational work. Further refinement and validation are recommended to strengthen its reliability as an appraisal tool.

**Implications:**

Findings highlighted a critical need to establish a standardised, validated approach to assess quality in action research. Adoption of QuARC can enhance consistency, clarity and comparability across studies, strengthening the evidence base for action research methodologies.

**Impact:**

This first systematic synthesis of QuARC's application provides an evidence base for its further development. This lays foundations for international standards in quality appraisal, strengthening the credibility, reproducibility and influence of action research.

## 1 Introduction

In traditional research, rigour refers to the systematic and disciplined application of methods. For quantitative research it specifically refers to the reliability and validity of the study. In qualitative research it is the trustworthiness, consistency and transparency of the research to ensure the credibility, dependability, confirmability and transferability of research findings. Reason and Bradbury (2008) suggest that the judge for quality action research be on the basis that it develops a praxis of relational knowledge, meaning the application of theory and knowledge generation reflects co‐operation between the researcher and participants. They use the term ‘quality’ in action research rather than rigour. Quality in action research refers not only to the circumstances that surround the study setting (physical, emotional, psychological, social and cultural contexts), but also must show how data are generated, gathered, explored and evaluated, how events are questioned, interpreted and evaluated in terms of workability through multiple action research cycles. However, the extent to which these dimensions are made explicit in published reports remains variable. It is increasingly important to appraise the quality of action research studies as their popularity continues to grow in healthcare settings and practitioners come to rely on the findings for implementation. Action research is frequently misjudged and seen as anecdotal and subjective and inherently biassed because the researcher, particularly if an insider, is sometimes perceived as lacking objectivity and independence (Casey et al. 2023). Indeed, these authors found that action research studies often lacked the specificity and details needed to assess the rigour or quality of the work and there often has been little evidence of application of any quality assessment frameworks in practice. This raises questions about how quality criteria are operationalised and reported across contemporary healthcare action research. This review systematically examines how published healthcare action research studies operationalise the Quality Assessment Action Research Checklist (QuARC) and evaluates its utility as a framework for quality appraisal.

## 2 Background

The concept of quality in action research is widely debated, with differing views on whether conventional quality criteria are appropriate for assessing such approaches (Spencer et al. 2003). The term ‘quality’ remains ambiguous, often used interchangeably with rigour, validity, trustworthiness, credibility and workability (Herr and Anderson 2015). This ambifuity has led to the development of multiple criteria, frameworks and checklists aimed at evaluating quality in action research. A brief synthesis of these is presented in Table 1.

**TABLE 1:** Summary synthesis of key quality action research frameworks.

Framework	Primary contributions	Limitations
Shani and Pasmore (1985)	Offers a balanced and systemic view of action research quality by integrating context, relationships, process, and outcomes. It is particularly useful for insider action research, where relational dynamics and organisational embeddedness are central.	Limited explicit attention as to how to operationalise quality within each domain. Participation and power, are more implicit rather than explicitly theorised.
Morrison and Lilford (2001)	Clarifies core methodological tenets of action research such as flexibility, iteration, and contextual sensitivity. The model is accessible and helpful for justifying action research in practice‐based and improvement‐focused studies.	Tends to privilege improvement outcomes over critical or theoretical contribution. Participation and power relations are assumed rather than foregrounded.
Levin (2003)	Strong emphasis on participation, joint meaning‐making and addressing real‐life problems. It legitimises practitioner and participant knowledge.	Less explicit focus on theory‐building and methodological rigour or to how local knowledge contributes to broader theoretical insight.
Eden and Huxham (2006)	This model provides one of the most methodologically rigourous accounts of action research, explicitly linking theory, action and reflection. It offers strong criteria for scholarly credibility and contribution beyond the immediate context.	Can be demanding and less flexible in emergent, participatory contexts. It can over emphasise academic priorities over participant‐led change.
Coghlan and Shani (2014, 2018)	Integrated, systemic approach to action research quality, balancing context, relational dynamics, process rigour, and outcomes; well suited to insider and organisational AR.	Broad categories require careful operationalisation by the researcher.
Bradbury (2025)	Contemporary framework foregrounding intentionality, reflexivity, sustainability and plurality of knowing.	As a values‐oriented and reflective framework it provides less procedural specificity, which may challenge researchers seeking concrete methodological guidance or assessment criteria.

These frameworks represent increasing efforts to define and assess quality in action research informing editorial and scholarly judgements. Earlier models emphasise structure, outcomes and rigour, whereas more recent frameworks foreground intentionality, reflexivity, sustainability and epistemic plurality. The central methodological challenge lies in selecting and justifying a framework that aligns with the study's context, modality, and purpose, rather than treating any single model as universally applicable. To date, there have been no studies synthesising the evidence on the quality of action research studies in healthcare. The Quality Action Research Checklist (QuARC) operationalises these traditions by translating them into four quality domains: context (Coghlan and Shani 2014), quality of relationships (Bradbury 2025; Levin 2003), quality of the action research process (Eden and Huxham 2006), and outcomes, encompassing both practical change and theoretical contribution (Coghlan 2019). In this way, QuARC provides a structured, transparent mechanism for enacting and evidencing core action research principles while preserving its participatory and emergent character.

### 2.1 The QuARC Framework

Drawing on Shani and Pasmore's (1985) work, Coghlan and Shani (2014, 2018) propose an action research framework informed by an extensive literature synthesis of published literature across a variety of organisations.

The framework comprises four quality domains:

- Context: The external and internal organisational environment including engagement with relevant scholarly literature informing the intended contribution.
- Quality of relationships: The extent to which relationships need to be managed, building trust, concern for the other, facilitating honest conversations, equality of influence in designing, implementing, evaluating the action and cogenerating the emergent practical knowledge.
- Quality of the action research process itself: The degree to which the inquiry is systematic, rigourous and reflexive, enabling deepened understanding of the issue.
- Outcomes: The generation of both practical and theoretical outcomes with consideration of sustainability and the development of participant capability
- Building on the work of Coghlan and Shani (2018), Casey et al. (2023) developed the Quality Action Research Checklist (QuARC) to support transparent reporting and objective appraisal of action research. QuARC is a brief 17‐item checklist organised around four quality domains—context relationships, process and outcomes (Shani and Pasmore 1985; see Table 2). To date, no reviews have synthesised evidence using QuARC as a reporting framework. This paper addresses this gap by synthesising the quality of published healthcare action research using QuARC to evaluate its utility through narrative analysis of the evidence on the quality of action research studies in healthcare.

**TABLE 2:** Quality Action Research Checklist (QuARC).

Factors	Questions
Context	Is the action driven by practical concerns? Are there local, national and international imperatives influencing the practical concerns? Does previous research inform the practical concerns? Has the AR project drawn on previous research?
Quality of relationships	Are those who own the practical concerns and have a stake in their resolution included in all the stages of the research project? Can the participants be classified as co‐researchers? Is the level of the patients/clients and co‐researchers evident at each stage of the research project? Is the AR project evaluated in terms of the quality of relationships?
Quality of the action research process itself	Does the final account demonstrate a clear and rigourous engagement in the AR project's design? Does the account demonstrate subsequent enactment of cycles of constructing, planning action, taking action and evaluating? Is the enactment of the cycles towards the practical and theoretical objectives transparent and reliable? Is the engagement of the co‐researchers evident in shared analysis and shared reflection?
Outcomes	Are both forms outcomes (theory/research and practical) presented? Are the outcomes sustainable? Are the outcomes useful for other AR projects? Does the theoretical outcome contribute to future theory development? Is there a reflection on the process of engagement on the cycles by the co‐researchers?

## 3 Methods

This study employed Turnbull's Turnbull et al. (2023) hybrid systematic narrative review methodology structured as a six‐stage sequential process comprising clarification of the research questions, justification of the approach, systematic identification of literature sources and search parameters, data cleaning, followed by information narrative synthesis and reported with relevant PRISMA 2020 guidance (Page et al. 2021). The approach includes the justification for the hybrid review and integrates systematic search protocols with explicit inclusion and exclusion criteria, followed by structured screening and critical appraisal using the QuARC tool, and narrative synthesis to interpret findings. The design enabled a comprehensive yet focused synthesis of healthcare action research to evaluate how quality is reported and to assess the utility of QuARC. The process included clarification of the research questions, justification of the methodology, systematic identification of literature sources and search parameters, data cleaning, followed by narrative synthesis. Thematic analysis was based on the four domains of QuARC to identify patterns, relationships, and gaps relevant to the two review objectives.

### 3.1 Aims and Research Questions

The aim of this study was to evaluate how published AR studies operationalise each QuARC item and identify recurring reporting gaps.

The research questions were as follows:

1. How do published studies identify and report on the quality of their studies using the 17 question items contained in QuARC?
2. Which of the 17 question items is least well addressed?

### 3.2 Literature Sources

1. Scopus was selected due to its comprehensive indexing of health sciences and nursing journals and its compatibility with bibliometric filtering through SciVal. Scopus was searched by two authors using the term ‘Action Research’ to locate health sciences articles. We excluded veterinary and dentistry and Health Professions from the search category because they were not the focus of the study. Midwifery articles were included as midwifery is part of the nursing category of Scopus.
2. To map the action research landscape, a bibliometric analysis of discipline‐spanning contributions to the field of action research in health sciences context was undertaken.
3. To select leading articles in these fields, the articles discovered in Scopus were exported to SciVal and the Field‐Weighted Citation Impact (FWCI) was applied. Only publications included in Scopus have a FWCI. It considers the differences in research behaviour across disciplines as it attempts to compare like with like by comparing an output's citation with those of other outputs for the same age and type classed by Scopus as being in the main subject area within this study is that of health sciences. The FWCI is the ratio of the document's citations to the average number of citations received by all similar documents over a 3‐year window. A FWCI of 1.00 indicates that the Publication Set's publications have been cited exactly as would be expected based on the global average for similar publications. The metric is always defined with a reference to a global baseline of 1.0 and intrinsically accounts for differences in citation accrual over time, differences in citation rates for different document types (e.g., reviews typically attract more citations than articles), as well as subject‐specific differences in citation frequencies overall and over time and document types. It is therefore a sophisticated normalised bibliometric (Purkayastha et al. 2019, 636). A threshold of FWCI > 1.00 was applied to focus the review on studies published in journals performing above the global citation baseline within health sciences. While not a standard criterion in systematic reviews, this threshold was used pragmatically to manage scope and is acknowledged as a potential source of citation bias.

### 3.3 Search Parameter Design

A systematic literature search strategy for Scopus was optimised with the assistance of a librarian (see Table 3). Once the articles were initially selected based on the authors' self‐identification of their work as action research studies, the following standard action research definition was added to provide some inter reviewer consistency in the interpretation and selection of action research studies.

… an emergent inquiry process in which applied behavioural science knowledge is integrated with existing organisational knowledge and applied to solve real organisational issues. It is simultaneously concerned with bringing about change in organisations and developing self‐help competencies in organisational members and adding to scientific knowledge. Finally, it is an evolving process that is undertaken in a spirit of collaboration and co‐inquiry. (Coghlan and Shani 2018: 4)

**TABLE 3:** Scopus search strategy.

Search term	‘Action research’ in Title and Author Keywords This approach was deliberate to broaden the inclusion of all studies labelled by the respective authors as being ‘action research’. As a result studies were selected which subsequently transpired not be action research studies when we applied the definition of action research by Coghlan and Shani (2018). This reduced the risk of selection bias.
Scopus categories	Health Sciences which includes Medicine, Nursing (includes midwifery) Veterinary, Dentistry and Health Professions

### 3.4 Eligibility Criteria

The inclusion and exclusion criteria are presented in Table 4.

**TABLE 4:** Inclusion and exclusion criteria.

Inclusion criteria	Exclusion criteria
Empirical studies from leading journals in the field of general Medicine, Nursing and Midwifery clinical contexts in which authors self‐identified their research article as an action research study	All other types of research studies or studies that lacked information and descriptions on the quality or rigour of action research. This exclusion criterion was adopted because the lack of information on the entire action research process would prevent the analysis of the application of QuARC.
Only English language and available in full texts	Literature reviews, commentaries, editorials and opinion pieces and articles in non‐clinical healthcare care contexts.
Peer reviewed empirical action research studies	Journals that publish less than twice a year and those that had ceased publication.
Time limits	January 2020–March 2024

### 3.5 Data Cleaning

All records identified through Scopus with a FWCI of greater than 1 were exported into Rayyan, a reference management software, and duplicate citations were removed prior to screening. Screening was conducted in two sequential stages: title screening and abstract screening, and full‐text review.

At the title and abstract screening stages, records were independently assessed for relevance against the predefined inclusion and exclusion criteria aligned with the review questions. Studies were retained where relevance could not be confidently determined from the title or abstract alone. Full texts of potentially eligible articles were then retrieved and assessed in detail against the eligibility criteria. Disagreements between reviewers were discussed and a third reviewer, who was not part of the original pairing, was consulted when needed to resolve the conflict until consensus was reached.

### 3.6 Information Synthesis

Data were synthesised and analysed using a process. Data were extracted independently by three authors using a bespoke Microsoft Excel and Microsoft Word template and verified by at least one other team member. Extracted data included study characteristics such as author, country, year of publication, aims and objectives, study design and type of action research, sample, data collection and analysis. The identification of QuARC factors was also undertaken and analysed in detail using a scoring system (Appendix 1) to capture each of QuARC's 17 quality items, which were scored as 0 (absent), 0.5 (partial), or 1 (comprehensive). To enhance consistency, three studies were independently pilot‐scored by all reviewers and discrepancies were discussed and resolved prior to full application of the tool. The scoring system enabled transparency in rating regarding evidence certainty between studies and raters; however, as there were different raters, the ratings were used as relative rather than absolute judgement as suggested by Cook and Reed (2015). No formal inter‐rater reliability statistics were calculated; therefore, scores should be interpreted as structured comparative indicators rather than definitive quantitative measures.

### 3.7 Data Analysis and Synthesis

Primary analysis involved extracting and aggregating scores for each of the 17 QuARC items, followed by domain‐level synthesis across the four quality factors. The scoring system also revealed inconsistencies and areas for improvement reported in tables.

## 4 Results

### 4.1 Study Characteristics

A total of 459 records were initially retrieved and reduced to 149 following application of the FWCI > 1 threshold. These were imported into Rayyan (Oussani et al. 2016) from EndNote (EndNote V.20.4.1) with 32 articles included in review (Figure 1).

**FIGURE 1:**
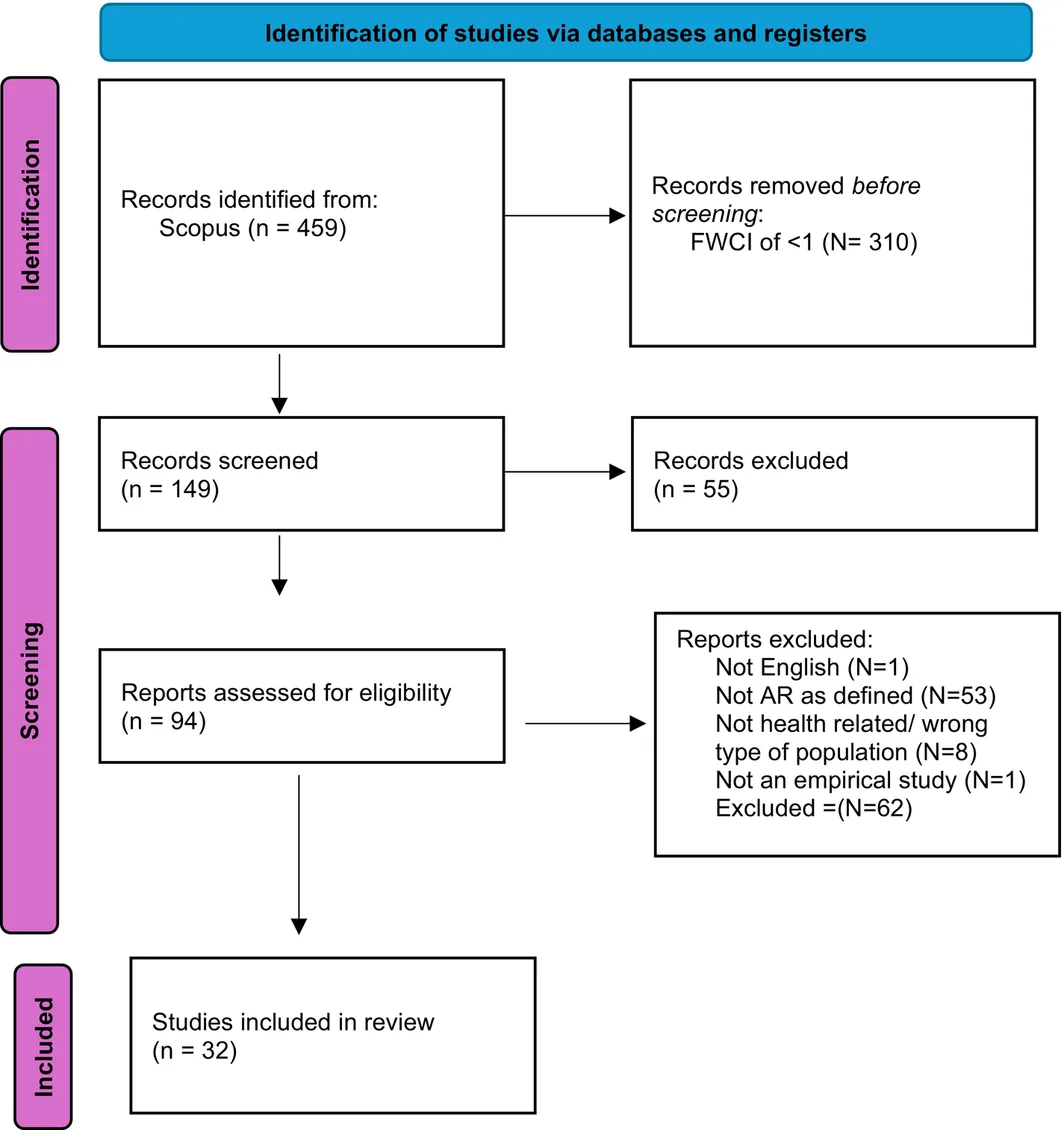
PRISMA 2020 flow diagram for new systematic reviews which included searches of databases and registers only.

The studies were from a broad geographical area including Australia (*n* = 4) (Armstrong et al. 2022; Cairns et al. 2022; Devlin et al. 2022; Gardner et al. 2023); Belgium (*n* = 1) (Karam et al. 2022); Brazil (*n* = 1) (Reis et al. 2023); Canada (*n* = 3) (Moussa et al. 2023; Doucet et al. 2022; Giesbrecht et al. 2023; Denmark (*n* = 1) Mikkelsen et al. 2022); England (*n* = 3) (Fenzi et al. 2022; Fogg et al. 2022; Gordon et al. 2022; Finland (*n* = 1) Jokiniemi et al. 2023); Ireland (*n* = 2) (Doherty and O'Brien 2022; Molloy and Phelan 2022); Netherlands (*n* = 3) (Bektas et al. 2023; De Jong et al. 2023; Lindenschot et al. 2023); New Zealand (*n* = 1) (Neville et al. 2022); Norway (*n* = 1) (Julnes et al. 2023; Pannekoeke et al. 2023); Nigeria (*n* = 1) (Arije et al. 2023); Pakistan (*n* = 1) (Moran et al. 2023); South Africa (*n* = 2) (Mabetha et al. 2023; Stuart‐Röhm et al. 2023); Southern Africa (*n* = 1) (Pittalis et al. 2023); Spain (*n* = 1) (Cabrera et al. 2023); Switzerland (*n* = 1) (Amati et al. 2022); Taiwan (*n* = 1) Chang et al. (2023); Thailand (*n* = 2) (Chaknum et al. 2023; Perngmark et al. 2023).

A summary of the characteristics of the included studies that addressed quality in action research is presented in Table 5 hereunder.

**TABLE 5:** Summary characteristics of included studies that addressed quality in action research.

References and region	Aim(s)	Study design	Sample and setting	Data collection and analysis	Key findings/relevance
Amati et al. (2022), Switzerland	Engage healthcare professionals in Lean process improvement to reduce surgical changeover time	Action Research using Lean (Gemba walks, SMED)	Interprofessional Operating Room team (nurses, surgeons, admin staff, cleaners)	Workshops (42 h), video analysis; SMED system	Improved collaboration enabled parallel processing; reduced OR turnover time
Arije et al. (2023), Nigeria	Co‐develop HIV prevention package for adolescent girls and young women	Participatory action research (PAR) with mixed methods, PDSA cycles	4308 for survey, 53 FGDs, 40 interviews, 3 sites	Surveys, FGDs, interviews; descriptive and thematic analysis	Five interventions co‐designed (parental comms, peer/facilitator‐led, condom outlets, social media); 77.5% reached
Armstrong et al. 2022, Australia.	Co‐create educational resources to support intercultural communication	PAR, 7 cycles	52 Yolŋu and Balanda adults; Northern Territory	Group and individual discussions; collaborative reflection	Resources resonated across Indigenous and non‐Indigenous groups; supported intercultural service provision
Bektas et al. (2023), Netherlands	Develop maternal/child health promotion action	PAR, iterative cycles	6 mothers in sessions; 9 interviewees	Online sessions, interviews; thematic analysis	Created ‘Mama's World Exercise Club’; empowerment and pride reported
Cabrera et al. (2023), Spain	Improve onco‐haematology patients' sleep	PAR, environmental intervention	18 professionals, 3 patients, 3 relatives; 318 patients (pre/post)	Group discussions; descriptive and inferential statistics	Reduced staff‐generated noise; further interventions needed
Cairns et al. (2022), Australia	Explore culturally responsive rehab service	Mixed‐methods Action Research	Stakeholder group (Indigenous + non‐Indigenous)	Yarning, informal conversations, stakeholder meetings; thematic analysis	Service redesign embedded reciprocity, holistic approach, Indigenous mentorship
Chaknum et al. (2023), Thailand	Develop family‐based care model for stroke survivors	PAR, 4 cycles	14 caregivers in two groups	Focus groups, observations, home visits, messaging app; ATLAS.ti thematic analysis	Four themes: problem assessment, collaboration, empowerment, healthy family dynamics
Chang et al. (2023), Taiwan	Develop nurse‐led shared decision‐making model for CAM in diabetes	PAR, 2 cycles, pre‐post	Interprofessional team 5–30 patients	Focus groups, questionnaires, clinical outcomes; thematic and statistical analysis	SDM and CAM understanding improved significantly; modest improvement in glucose
De Jong et al. (2023), Netherlands	Examine coalition processes in health promotion	PAR within community coalition project	Coalition of 6 organisations and health broker	CAC and CNA frameworks, interviews, group sessions; thematic + network analysis	Insights into sustaining intersectoral collaboration over 2 years
Devlin et al. (2022), Australia	Stop TB transmission in large Aboriginal family household	PAR integrating housing, nutrition, TB care	A father and 14 family members	Informal discussions, debriefing	Enabled early TB diagnosis, treatment, and prevention through housing and nutrition interventions
Doherty and O'Brien (2022), Ireland	Explore midwives' understandings of burnout	PAR with co‐operative inquiry	21 practising midwives, 3 students, Ireland	Five inquiry meetings; thematic network analysis	Multiple factors influenced perceptions of burnout; insights into personal and professional impact
Doucet et al. (2022), Canada	Examine relationships that matter for youth ageing out of care	PAR using photovoice	8 youth with lived experience and 3 researchers	12 weekly group sessions, photography training, photo analysis	Promoted healing, empowerment, and political agency; informed child protection policy
Fenzi et al. (2022), England	Identify training needs of new emergency nurses, pilot simulation training	Action Research in 3 phases	Convenience sample of emergency nurses	Focus groups; transcripts analysed via Colaizzi	Proposed simulation‐based training content; positive perceptions of MAES method
Fogg et al. (2022), England	Develop/evaluate community respiratory care model (MISSION ABC)	PAR	Patients, clinicians, research staff, ethicists	Meetings, reflections, outcome evaluation	Highlighted benefits/challenges of PAR; ethics and governance issues noted in trial design and consent
Gardner et al. (2023), Australia	Enhance clinical supervision framework for allied health	Action Research, 4 cycles over 5 years	11 allied health managers in regional service	11 face‐to‐face meetings; qualitative content analysis	Six themes identified: implementation, framework, purpose, characteristics, discipline differences, support needs
Giesbrecht et al. (2023), Canada	Explore grief experiences of community service workers	Community‐based PAR	Primary participants: 18 service workers; Secondary: 48 across care sectors	Focus groups (5), interviews (13), field notes; interpretive thematic analysis	Workers experienced layered, compounded grief; findings support tailored grief supports
Gordon et al. (2022), UK	Develop relationship‐centred care in stroke units	Appreciative AR	Staff (*n* = 65), patients (*n* = 17), relatives (*n* = 7)	Observations, discussions, interviews; collaborative + researcher‐led analysis	Supported culture change towards valuing human connections; three processes enabled relational care
Jokiniemi et al. (2023), Finland	To describe the clinical nurse specialist role and its outcomes and challenges	Participatory action research	Multiple data collection methods	Enhancing the Quality and Transparency of health Research (EQUATOR) guidelines for participatory action research	Increased visibility of nursing expertise, development, integration and quality assurance of nursing processes and practice, and promotion of knowledge translation and unit and collaborator cooperation. Furthermore, role challenges were also recognised
Julnes et al. (2023), Norway	To explore nurses' use of the afferent limb of the Rapid Response System to recognise and respond to deteriorating patients	Action research	Systematic text condensation. COREQ	Video recordings with observations of 20 registered nurses	Customised education programmes and fidelity‐scale simulations are suited but not sufficient to change clinical competency without management anchoring
Karam et al. (2022), Belgium	To assess IPC between general practitioners (GPs) and nurses; identify target priorities for improving IPC; and facilitate the planning and implementation of the proposed improvement strategies	Participatory action research	Meetings to examine intent, experiences, expectations, and fears	SWOT analysis of IPC through a deductive thematic analysis approach	Mutual lack of knowledge between colleagues and how each functions professionally, and their personal constraints and needs identified
Lindenschot et al. (2023), Netherlands	Optimise video‐based PRPP‐Assessment for children with mitochondrial disorder	Action design research	Parents of affected children + Radboud UMC staff	Logs, feedback, interviews; content analysis	Lessons in instructing parents, handling videos, assessing limited‐function children
Mabetha et al. (2023), South Africa	Analyse community power‐building in PHC, develop participation guidance	PAR	3 rural villages, service users and providers	Workshops, notes, visual/VA data, stakeholder feedback; thematic analysis	Co‐construction of evidence built collective capabilities for PHC participation
Mikkelsen et al. (2022), Denmark	Explore patients' perspectives on living with abdominal aortic aneurysm (AAA)	Action Research	Purposive sample, vascular outpatient clinic	Observations, interviews, workshop	Identified psychosocial impacts of AAA; development of supportive mobile application
Molloy and Phelan (2022), Ireland.	Understand palliative care meaning for staff in long‐term care	Co‐operative inquiry AR	18 Health Care Assistants and 16 nurses	Group discussions of cases; thematic analysis	Three themes: Living, Loving, Letting go; reflected staff experiences of end‐of‐life care
Moran et al. (2023), Pakistan	Co‐design COVID‐19 risk communication strategy in rural community	PAR, 2 groups	30 participants (male/female groups), 500‐household community	Group discussions; thematic analysis	Nine themes including faith, vaccination attitudes, social impacts, women's empowerment
Moussa et al. (2023), Canada	Develop education manual for SCI patients in Gaza, via occupational justice lens	PAR	54 participants (focus groups with 10–12 people in each)	8 FGDs; inductive thematic analysis	Themes: enabling occupational justice, removing barriers
Neville et al. (2022), New Zealand	Identify barriers to older Pacific people's healthcare participation	PAR	104 older people	Focus groups using Talanoa thematic analysis	Barriers: access, provider relationships, health system understanding
Pannekoeke et al. (2023), Norway	Describe the initiation of CPD to develop simulation based learning (SBL)	PAR	10 university participants and 44 national simulation conference participants	Four workshop sessions using 40 evaluation forms. Qualitative content analysis	Two themes: (1) Culture and structure for professional development in SBL (2) The development of competence and confidence in facilitation
Perngmark et al. (2023), Thailand	Develop implement and evaluate Islamic family care programme for Thai‐Muslims	AR design	5 community hospitals. Purposive sampling of 17 adults with chronic illness	Semi structured interviews, discussions and observations. Constant comparison analysis	Themes: Inadequate knowledge, insufficient skills, low family involvement, poor negotiation skills, and the need for better integration of Islamic doctrines
Pittalis et al. (2023), Southern Afric	To empower district hospitals to deliver safe essential surgery via PAR	Participatory action	District surgical teams and stakeholders, 31 hospitals across 3 countries	Workshops, group discussions, notes; thematic analysis	Identified training/supervision gaps; co‐designed tailored training; strengthened local ownership
Reis et al. (2023), Brazil	To improve inpatient bed setup using lean practices with AR	Action Research; 5‐stage cycle (diagnosis–learning)	One public hospital (139 beds)	Interviews, ERP data, observations, meetings; VSM, kaizen.	Empowered frontline staff; reduced bed setup time; boosted capacity without extra resources.
Stuart‐Röhm et al. (2023), South Africa	Assess acceptability and caregivers' experiences of person‐centred caregiver singing (PCCS)	Action research	39 Care‐home caregivers	Music‐therapy‐infused sessions; thematic content analysis	Positive impacts on residents' wellbeing, caregiver capability and empathic connection; implementation hindered by limited song repertoire and resident unpredictability

### 4.2 Quality Appraisal

All included studies addressed at least one QuARC quality domain. This was expected as inclusion criteria required explicit reference to quality‐related elements. Nevertheless, the frequency of reporting varied between factors as seen in Figure 2. Reported frequencies varied across domains: Context factor was 92.5%, mean = 3.7/4, Quality of Relationships (55% mean = 2.2/4), Quality of AR Process (62.5% mean = 2.5/4), and Quality of Outcomes (62.5% mean = 3.1/5). A small number of studies did not clearly describe enacted action cycles.

**FIGURE 2:**
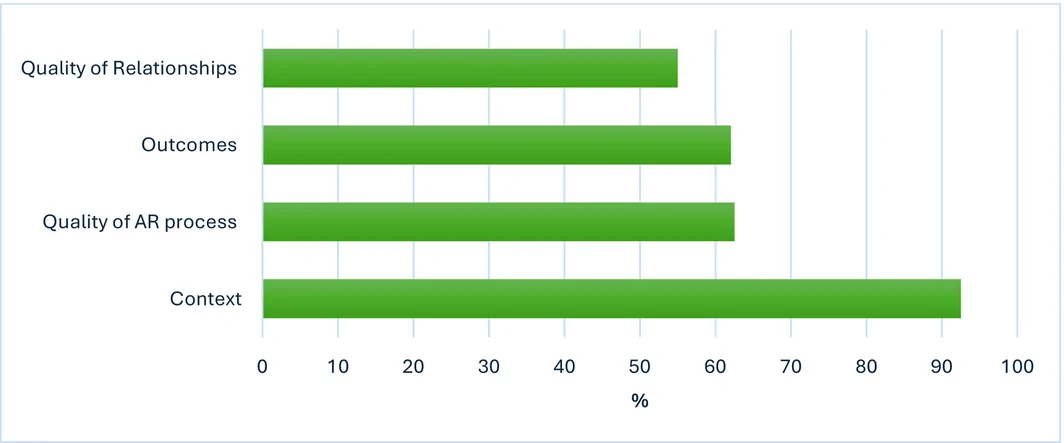
Frequency of reporting QuARC factors.

### 4.3 Research Question 1. Reporting on Quality Factors Using QuARC

#### 4.3.1 QuARC Factor on Context

##### 4.3.1.1 Is the Action Driven by a Practical Concern?

All 32 studies were grounded in identifiable practice concerns, although the degree of contextual depth varied. Most problems originated in clinical practice, service delivery, or community health needs. For example, Jokiniemi et al. (2023) addressed uncertainty surrounding the role of Clinical Nurse Specialists a workforce issue with implications for service redesign. Julnes et al. (2023) focused on improving nurses' use of rapid response systems, while Lindenschot et al. (2023) looked at informed consent practices in paediatric physiotherapy. Although many concerns were practice‐based, stakeholder involvement in defining the initial problem varied across studies. In contrast, Molloy and Phelan (2022) explicitly engaged older people in shaping care priorities, and Mikkelsen et al. (2022) addressed communication gaps in vascular nursing.

##### 4.3.1.2 Is There a Local, National or International Imperative Influencing the Practical Concern?

Most studies situated their practical concerns within wider policy or system‐level imperatives. For example, Chaknum et al. (2023) linked their study to evidence supporting shared decision‐making models for complementary and alternative medicine disclosure. Moran et al. (2023) framed their work in relation to global knowledge translation challenges in low‐ and middle‐income countries. These connections strengthened the relevance of the action research beyond the immediate setting.

##### 4.3.1.3 Does Previous Research Inform the Practical Concern?

Engagement with prior research was evident in several papers. Bektas et al. (2023) drew on previous research to support the rationale for the inclusion of parents where it was found that interventions which have been co‐created with parents are more likely to lead to more acceptable and appropriate interventions. Other studies, including Julnes et al. (2023) and Lindenschot et al. (2023), foregrounded local problem identification with more limited engagement with the wider evidence base.

##### 4.3.1.4 Has the AR Project Drawn on Previous Research?

Engagement with previous research was evident in several papers. Arije et al. (2023) drew on previous action research projects that targeted youths and their communities with HIV prevention programming. Likewise Chaknum et al. (2023) drew on previous research to show that families of stroke survivors face challenges in daily life that can involve heightened anxiety, depression, stress, and poor social adjustment. Similarly, Cabrera et al. (2023) positioned their work within a body of research advocating non‐pharmacological approaches to improving sleep in critical and complex care settings.

##### 4.3.1.5 Summary

Overall, the reviewed studies demonstrated a strong orientation towards practice‐driven problems, often situated within clear local, national or international imperatives. However, the quality of contextualisation varied markedly. While some studies explicitly integrated prior empirical and theoretical work to inform both the articulation of the problem and the design of the action research, others relied primarily on locally perceived needs with limited engagement with the wider evidence base. The degree of conceptual grounding and explicit engagement with prior literature varied across studies. These contextual variations have important implications for how stakeholders are identified, engaged and positioned within action research processes, which is examined in the following QuARC factor.

#### 4.3.2 QuARC Factor on Quality of Relationships

##### 4.3.2.1 Are Those Who Own the Practical Concern Included in All Steps of the AR Cycle?

Full‐cycle involvement of stakeholders was reported by Mabetha et al. (2023) where community members helped define the issue, co‐designed responses and reflected on progress through structured workshops. In contrast, Moran et al. (2023) reported the use of feedback loops but lacked detail on how decisions were co‐negotiated, and Molloy and Phelan (2022) did not elaborate on how the voices of older adults were integrated throughout the project.

##### 4.3.2.2 Can the Participants Be Classified as Co‐Researchers?

Many studies referred to participants as co‐researchers (Arije et al. 2023; Bektas et al. 2023; Cairns et al. 2022; Chaknum et al. 2023; De Jong et al. 2023; Devlin et al. 2022; Amati et al. 2022). Mabetha et al. (2023), named community actors as partners in knowledge co‐production while Jokiniemi et al. (2023) framed practitioners as equal contributors, though the term ‘co‐researcher’ was not consistently used. Doucet et al. (2022) explicitly adopted co‐researcher terminology.

##### 4.3.2.3 Is the Level of Participation of the Clients/Patients/Co‐Researchers Evident at Each Step?

Several studies mentioned the level of participation of participants as co‐researchers with Stuart‐Röhm et al. (2023) and Gordon et al. (2022) highlighting ongoing reflexive conversations on how the researcher and co‐participants were ‘going on together’ by exploring each other's roles in the process of creating action.

##### 4.3.2.4 Is the AR Project Evaluated in Terms of the Quality of the Relationships?

Only a few studies explicitly evaluated relational quality (Bektas et al. 2023; De Jong et al. 2023; Pittalis et al. 2023; Stuart‐Röhm et al. 2023). Mabetha et al. (2023) provided the most developed example, using a power‐oriented framework to examine trust, participation, and shared control. Moussa et al. (2023) describe relational depth through collaborative activities with co‐researchers and analysed data to identify core components of the education tool including its content, format, and use in rehabilitation services.

##### 4.3.2.5 Summary

This section examined the extent and depth of stakeholder involvement in Action Research (AR) studies. Some studies demonstrated full participation, with stakeholders involved in defining issues, co‐designing actions and reflecting on progress. Others provided limited detail on how decisions were shared or how participant voices were sustained throughout. Many studies described participants as co‐researchers or equal partners in knowledge co‐production, although terminology was not used consistently. In some cases, reflexive dialogue and shared roles were clearly articulated, indicating collaborative engagement across stages of the AR process. That said, only a few studies explicitly evaluated the quality of relationships. Where assessed, attention was given to trust, power‐sharing, participation and shared control.

#### 4.3.3 QuARC Factor on Quality of the Action Research Process Itself

##### 4.3.3.1 Does the Account Demonstrate a Rigourous and Collaborative Engagement in the Action Research Project's Design?

This question item was extensively discussed. Arije et al. (2023) tested the potential intervention models to be included in the change package using the Plan‐Do‐Study‐Act (PDSA) cycle. Doucet et al. (2022) demonstrated how the research team members cooperated closely in all the study steps. Fogg et al. (2022) recount how data were collected in clinics from patients and carer participants who were engaged as researchers.

##### 4.3.3.2 Does the Account Demonstrate Subsequent Enactment of Cycles of Constructing, Planning Action, Taking Action and Evaluating?

Many studies provided clear evidence of iterative action research cycles linking problem construction, planning, action and evaluation (Amati et al. 2022; Arije et al. 2023; Armstrong et al. 2022; Bektas et al. 2023; Cairns et al. 2022; Chang et al. 2023; De Jong et al. 2023; Devlin et al. 2022; Fenzi et al. 2022; Giesbrecht et al. 2023; Molloy and Phelan 2022). For example, De Jong et al. (2023) described a participatory design process in which stakeholders contributed to network development and jointly reviewed findings. Doucet et al. (2022) provide a diagram showing how cycles of reflection, planning, action and analysis were enacted while Gordon et al. (2022) link the action‐reflection cycles with the appreciative inquiry cycle. Others articulated staged processes linking diagnosis, implementation, monitoring and evaluation (Perngmark et al. 2023; Reis et al. 2023).

##### 4.3.3.3 Is Enactment of the Cycle Towards the Practical and Theoretical Objectives Transparent and Reliable?

This was extensively addressed by Arije et al. (2023); Bektas et al. (2023); Cairns et al. (2022); De Jong et al. (2023); Fenzi et al. (2022); and Giesbrecht et al. (2023). Practical outcomes were well articulated, including changes to practice orientations (Gordon et al. 2022) and the co‐production of tangible resources such as the Spinal Cord Injury Activities of Daily Living Education Manual (Moussa et al. 2023).

##### 4.3.3.4 Is the Engagement of the Coresearchers Evident in Shared Data Analysis and Shared Reflection?

Many studies addressed this item extensively. Stuart‐Röhm et al. (2023) and Gordon et al. (2022) report that at the end of the project, meetings were held with representatives from the different groups of participants to discuss overall views about the study.

##### 4.3.3.5 Summary

Overall, studies commonly described iterative cycles of planning, action, and reflection, with varying levels of explicit linkage to theoretical knowledge generation. Studies frequently employed cycles of problem construction, planning, action, and evaluation, often using frameworks such as PDSA or appreciative inquiry, with stakeholder participation evident in co‐design, data collection, and analysis. Practical outcomes, such as changes to clinical practice and co‐produced resources, were generally clear and well‐documented, while theoretical contributions around knowledge of enablers and barriers were less consistently articulated. Coresearcher engagement in shared reflection and data analysis was commonly reported, though some studies provided only general accounts, limiting transparency of reflexive learning.

#### 4.3.4 QuARC Factor on Quality Outcomes

##### 4.3.4.1 Are Both Forms of Outcomes Presented (Theory) and Action (Practical)?

Just over half of the studies clearly articulated both action and theory outcomes. Several linked practice change with conceptual development, such as Gordon et al. (2022), who reported practice‐level intervention outcomes alongside theoretical extensions to lifeworld‐led and relational care, and Stuart‐Röhm et al. (2023), who developed a Person‐Centred Caregiver Singing Model capturing interdependent relational and ecological effects. Reis et al. (2023) combined operational improvements with theoretical contributions to bed management and lean methodologies. In contrast, other studies focused primarily on practical learning or implementation outcomes, with limited explicit theorisation (e.g., Giesbrecht et al. 2023; Pannekoeke et al. 2023).

##### 4.3.4.2 Are the Outcomes Sustainable?

Sustainability was explicitly addressed in just over half of the studies. Evidence included the transfer of interventions to new settings (Bektas et al. 2023). Devlin et al. (2022), showcased how lessons learned could inform future action research projects across related contexts, demonstrating how quality outcomes contribute to a cumulative knowledge base. Doucet et al. (2022) made the case that posting their model and a summary of the outcomes on the university website ensured its sustainability.

##### 4.3.4.3 Are the Outcomes Useful for Other Action Research Projects?

A substantial majority of studies demonstrated relevance for other action research contexts, often through methodological reflection or transferability considerations. For example, De Jong et al. (2023) explicitly distinguished between context‐specific and potentially generalisable processes, supporting use of findings in future practice‐based research.

##### 4.3.4.4 Does the Theoretical Outcome Contribute to Future Theory Development?

Fewer than half of the studies clearly positioned their findings as contributions to ongoing theory development. Where this was addressed, authors highlighted learning related to intercultural communication, reciprocity, and professional–practice tensions (Armstrong et al. 2022; De Jong et al. 2023), or identified areas requiring further conceptual exploration, such as divergent understandings of occupational justice (Gordon et al. 2022).

##### 4.3.4.5 Is There Reflection on the Process of Engagement and the Cycles by the Co‐Researchers?

A quarter of the studies provided detailed accounts of co‐researcher reflection on engagement and cyclical processes. (Moussa et al. 2023; De Jong et al. 2023; Stuart‐Röhm et al. 2023; Jokiniemi et al. 2023; Mabetha et al. 2023). Strong examples included collaborative synthesis of participant data and researcher reflections (Armstrong et al. 2022) and explicit acknowledgement of partial or constrained participation within PAR processes (Moussa et al. 2023). Moussa et al. (2023) offered more normative conclusions of what ought to be without reflexive evaluation.

##### 4.3.4.6 Summary

Just over half of the studies addressed both action and theory outcomes. However, the sustainability of outcomes was explicitly addressed in just over half of the studies, most commonly through transfer of interventions to new settings or through institutional dissemination mechanisms. In contrast, the majority of studies demonstrated usefulness for other action research projects, particularly by distinguishing between context‐specific and transferable processes. Fewer than half clearly articulated contributions to future theory development. Reflection by co‐researchers on engagement and cyclical processes was evident in only a minority of studies.

### 4.4 Cross Study Patterns Across QuARC Factors

As seen in Table 6, across the reviewed studies, the contextual quality factor was consistently strong, with all action research projects driven by identifiable practice concerns and frequently situated within local, national, or international imperatives. However, there was a pattern of emphasising more contextual relevance rather than genuine participatory problem framing. In relation to the quality of relationships, participatory intent was frequently asserted, yet often in a formulaic manner that appeared oriented towards legitimacy or presentation rather than ensuring deep, sustained, or structural engagement. QuARC analysis revealed considerable variability in the extent to which those owning the practical concern were involved across all stages of the action research cycle. The quality of the action research process itself was a relative strength across studies. Iterative cycles of planning, action and reflection were commonly articulated, and stakeholder engagement in data collection and analysis was frequently reported. Nevertheless, shared reflection was often generalised, limiting transparency around reflexive learning across cycles. A clear pattern emerged in that practical outcomes were prioritised over theoretical contribution, with fewer than half explicitly positioning findings as contributions to ongoing theory development. Sustainability was inconsistently addressed and most often demonstrated through replication or dissemination rather than long‐term embedding.

**TABLE 6:** Results of the scoring of the QuARC individual items.

Factors	No	Questions	Score
Context	1	Driven by a practical concern	32
	2	Local, national and international imperative influencing the practice concern	32
	3	Previous research informed the practical concern?	32
	4	Previous research informed the practice concern	31
Quality of relationships	1	All who own the practice concern included in all steps of action research	24
	2	Participants classified as co‐researchers	23
	3	Level of participation of the patients/clients is articulated	15
	4	Evaluated quality of relationships	15
Quality of action research process itself	1	Demonstrates rigourous and collaborative engagement in the project design	23
	2	Demonstrates cycles of constructing, planning, acting and evaluation	29
	3	Enactment of cycles towards objectives transparent and reliable	25
	4	Engagement of co‐researchers in shared data analysis	21
Outcomes	13	Theory and practical outcomes presented	24
	14	Outcomes are sustainable	30
	15	Outcomes useful for other action research projects	29
	16	Theoretical outcome contributes to future theory development	18
	17	Reflection on the process of engagement	12

Conversely, transferability to other action research contexts was widely considered, with many studies distinguishing between context‐specific and generalisable processes. Moreover, reflexive evaluation by co‐researchers represented the weakest and least consistently addressed quality dimension. The pattern of the review revealed that only a minority of studies provided detailed accounts of collaborative reflection on engagement and cycles, with several relying on normative conclusions rather than analytic reflexivity. This pattern limits assessment of outcome validity and constrains cumulative knowledge generation. Essentially, the reviewed studies demonstrate that quality in action research is multi‐dimensional, requiring attention not only to the methodological soundness of processes but also to the authenticity of engagement, the responsiveness of inquiry to its context and the reflexive evaluation of its outcomes.

### 4.5 Research Question 2. Primary Analysis Related to the Identification of the 17 Question Items Included in QuARC

As summarised in Figure 3, gaps in reporting were concentrated in relational, process and outcome quality factors rather than in contextual grounding. Of the 17 items, only one related exclusively to contextual grounding, whereas the remaining items addressed relational, process and outcome dimensions. Across the dataset, items requiring explicit reflection on engagement across cycles and critical evaluation of relationship quality were least frequently addressed. The item relating to the outcome factor on the contribution to future theory was missed out by 14 studies, and the item relating to ‘is the engagement of the co‐researchers evident in shared data analysis and shared reflection?’ was not extensively addressed by 11 studies. This finding indicates that many studies prioritised practical change over knowledge building and reflexive collaboration. This suggests that outcomes were often reported as context‐bound improvements rather than positioned within broader conceptual or theoretical debates. Similarly, the limited attention to shared data analysis and reflection in 11 studies implies that, while participation may have occurred in practice, it was not consistently made visible at the analytical level, thereby weakening claims to co‐production, outcome validity and cumulative learning in action research.

**FIGURE 3:**
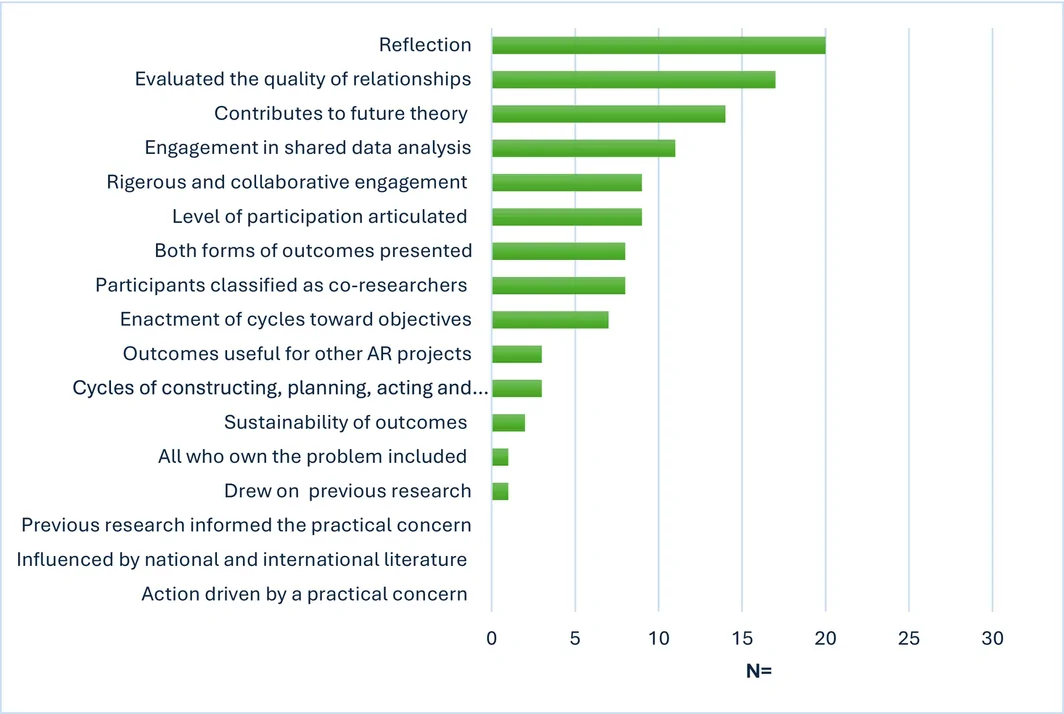
Items for Improvement in demonstrating quality in Action Research Studies.

## 5 Discussion

Aside from the QuARC, no other established tool currently exists for the critical appraisal or quality assessment of action research. Findings from this review provide empirical support for its use, demonstrating the value of its four interrelated quality factors in promoting methodological transparency and meaningful stakeholder engagement across the action research cycle. QuARC's explicit focus on context is a key strength as problems are framed, prioritising concerns and recognition of imperatives all shape legitimate stakeholder positioning and relationships within the research process. This highlights the interdependence of QuARC's quality factors. The following is a discussion on the review findings across the four QuARC factors.

### 5.1 QuARC Factor Context

The prominence of context in the reviewed studies aligns with literature highlighting its foundational role in shaping the relevance, legitimacy, and transferability of action research (Herr and Anderson 2015; Bradbury 2025). In action research, understanding the socio‐cultural, institutional, and practical settings in which problems arise is critical to generating actionable and contextually grounded knowledge (Coghlan and Brydon‐Miller 2014) and the review supports this with strong examples from Bektas et al. (2023) and Mabetha et al. (2023). In both cases, contextual awareness anchored the research in pressing real‐world concerns and strengthened its practical significance. Our findings highlight variability in how context is theorised and evidenced. As previous scholarship notes, rigourous action research depends on reflexive engagement with prior work and the explicit theorisation of context to ensure both methodological coherence and practical impact (Reason and Bradbury 2008; Coghlan 2019). While some studies demonstrated reflexive engagement with prior research and methodological literature, others foregrounded local problem identification with limited conceptual grounding (e.g., Karam et al. 2022; Mikkelsen et al. 2022). This unevenness suggests that despite widespread recognition it is not consistently operationalised or critically examined a gap QuARC addresses by requiring explicit attention to contextual drivers, imperatives, and evidence bases, thereby supporting more consistent and transparent evaluation of contextual quality.

### 5.2 QuARC Factor on Quality of Relationships

QuARC highlights that a defining feature of action research is its emphasis on collaboration and the quality of relationships between researchers and participants. The findings of this review support this emphasis, demonstrating that relational quality underpins genuine collaboration and the co‐creation of actionable knowledge. This focus aligns with the literature, which identifies the relational dimension as central to action research quality (McNiff and Whitehead 2001; Bradbury 2025; Reason and Bradbury 2008; Casey et al. 2023). Bektas et al. (2023), illustrate how relational integrity can be operationalised ethically and methodologically, strengthening the integration of emic and etic perspectives. This helps to distinguish action research from more traditional qualitative approaches (Coghlan and Brydon‐Miller 2014; Zuber‐Skerritt 2011). Although QuARC foregrounds relational quality as a dimension of rigour, many reviewed studies adopted the language of collaboration or co‐research without clearly demonstrating how relationships were negotiated, sustained or critically examined. This pattern echoes broader critiques that relational and practice‐based dimensions of action research are often marginalised within academic contexts that privilege theoretical abstraction, contributing to its mischaracterisation as participatory qualitative inquiry rather than research conducted with practitioners (Bradbury‐Huang 2010; Schein 2010). Overall, analysis of this QuARC factor indicates that while QuARC provides a robust framework for evaluating relational quality, its effectiveness is contingent on transparent and reflexive reporting though additional methodological guidance may be needed to support more consistent articulation of relational work in published action research.

### 5.3 QuARC Factor Quality of the Action Research Process

Quality in action research is grounded in the integrity of the process, specifically the extent to which collaboration, reflection and action are systematically, transparently and ethically enacted throughout all stages of the research cycle (Bradbury 2025; Coghlan 2019; Reason and Bradbury 2008; Morrison and Lilford 2001). Our findings demonstrated process integrity by enabling participants to decide their level of involvement in data analysis (Bektas et al. 2023), while Gordon et al. (2022) relate linking the action reflection cycles with the appreciative inquiry cycle describing their attention to how data were analysed. Doucet et al. (2022) also provide a diagram showing how cycles of reflection, planning, action and analysis were enacted. These findings align with what Herr and Anderson's (2015) term process validity, which focuses on the extent to which the research process itself generates new understanding and transformative action.

The third step of the action research cycle namely, that of ‘taking action’ may well involve collecting data as it is happening during the action research process to provide new and unexpected insights leading to new knowledge or emergent theory. It is possible that this emergent process also adds to the impression that the research process in action research is simply ‘a make‐up‐as‐you‐go‐along process’. Therefore, the inductive element of theory development in action research is often underappreciated.

Some studies in our review demonstrated theoretical outcomes in terms of contribution to knowledge which can be seen in communication and collaboration created by the PAR process and shared knowledge about enablers and barriers to occupational justice (Moussa et al. 2023). However, not all studies clearly linked cyclical action to knowledge generation, limiting assessment of theoretical contribution. Engagement of co‐researchers in shared data analysis and reflection essentially closes the loop on full participation, save perhaps for their possible inclusion in publications. A poor understanding of this quality factor could contribute to the thinking that action research is inherently unscientific and while the emergent and unfolding element highlights the complexity of ‘planning step’ in action research, it can be misinterpreted as being a haphazard process thereby losing its distinct epistemological and transformative intent. Therefore recognising the quality of the process itself including how relationships, cycles of reflection and action, and contextual responsiveness are managed as identified in QuARC ensures that action research remains a rigourous and credible approach to generating co‐created, actionable knowledge.

### 5.4 QuARC Factor Outcomes

The QuARC framework's emphasises that quality in action research arises not from procedural rigidity but from process transparency, reflexivity, and coherence between action and outcome. As the dual objective of action research is to take action as well as generate theory it is sine qua non that any quality criteria for action research cannot be separated from the dual objectives that define the approach: producing both actionable change and theoretical insight. Examples of dual‐outcome studies include Gordon et al. (2022); Stuart‐Röhm et al. (2023); and Reis et al. (2023), which explicitly articulated both practice‐based results and contributions to conceptual understanding. Casey et al. (2023) Reason and Bradbury (2008) and Herr and Anderson (2015) clearly attest that action research must address both dimensions—demonstrating practical outcomes that improve practice and theoretical outcomes that contribute to knowledge. This echoes findings by Zuber‐Skerritt (2011) that rigourous action research integrates both the practical and intellectual cycles of learning. The reviewed studies tended to privilege practical problem‐solving over theory development, revealing an imbalance that constrains action research's contribution to broader theoretical and methodological advancement. Viewed through the lenses of outcome validity (Herr and Anderson 2015) and Bradbury's (2025) theory–practice choice point, this pattern suggests that while tangible improvements were commonly achieved, opportunities to translate action into explicit theoretical insight were less consistently realised.

A critical factor of the QuARC outcomes factor involves sustainability of outcomes and hence the transferability and utility of findings beyond the immediate setting (Casey et al. 2023; Coghlan and Brydon‐Miller 2014). Sustainability, a choice point also highlighted by Bradbury (2025), is an increasingly vital marker of research quality in the current era of global uncertainty and was emphasised in the review by Amati et al. (2022) and Cairns et al. (2022), whose cyclical approaches demonstrated ongoing refinement and continuity across successive iterations of the research process. As Zuber‐Skerritt (2011) notes, such iterative learning represents both the hallmark and the test of quality in action research, ensuring that findings are not merely context‐specific but theoretically generative. The QuARC item of specifying the utility of the outcomes for ‘future action research projects only’, necessitates clarity. It is possible that this aspect of QuARC needs refinement to include all future research in general rather than on action research itself.

In relation to ‘reflection on the process of engagement and the cycles by the co‐researchers,’ this review found that relatively few studies provided explicit accounts of reflective evaluation. For example, Bektas et al. (2023) described shared and documented reflection among participants regarding their levels of engagement and the support needed to take action—illustrating good practice in reflexive learning. This quality dimension is crucial not only for ensuring learning within a project but also for generating rigourous and credible outcomes that can inform both theory and practice. Hence, while the value of action research as a process of developing a workable and sustainable solution to a given situation may be commended, it's meandering journey which captures the reality of the situation, may be seen as a less reliable and valid and consequently a less scientific way of creating knowledge. As Herr and Anderson (2015) observe, the emergent and adaptive nature of action research often challenges traditional positivist expectations of fixed design and predictability. Yet, this flexibility represents, as Reason and Bradbury (2008) argue, an epistemological strength rather than a weakness. Knowledge in action research is contextually emergent—it develops through cycles of action, reflection, and learning that mirror the complexity of real‐world practice. Such responsiveness ensures that outcomes remain relevant, authentic, and sustainable, thereby enhancing the quality of the inquiry process (Bradbury 2025; Zuber‐Skerritt 2011). As Bradbury‐Huang (2010) and Coghlan (2019) note, rigourous action research demonstrates its quality through evidence of thoughtful engagement, documented learning, and a clear link between practice‐based outcomes and theoretical insight. In this regard, QuARC provides a valuable structure for recognising reflection as both a methodological requirement and a key determinant of outcome quality, affirming action research as a credible and transformative approach to knowledge creation.

### 5.5 Critical Appraisal of QuARC

This review reveals that overall, the QuARC framework not only provides a robust framework for assessing action research quality but also makes visible structural patterns in the literature that constrain the epistemological and transformative potential of action research in the health sciences. The application of the QuARC framework in this review demonstrates a field that is methodologically confident in doing action research but less consistent in articulating its epistemic and relational foundations. This pattern suggests that while QuARC effectively captures strengths in action research practice, its application also reveals systematic gaps particularly in theory development, sustainability articulation and critical reflection on participation. QuARC enabled the demonstration of process rigour and responsiveness to contextual demands, countering claims that action research lacks methodological discipline. It also revealed that contemporary health‐related action research prioritises practical problem‐solving and local relevance while under‐developing its potential for theory advancement, relational accountability, and cumulative learning which are precisely the dimensions QuARC is designed to make visible.

The four QuARC quality factors of context, relationships, process and outcomes provide a coherent checklist for understanding and establishing quality in action research. The QuARC framework usefully operationalises these principles by making visible how each factor contributes to quality and by emphasising their interdependence. The QuARC's articulation of quality across these interconnected factors repositions quality as a living, participatory construct that reflects the epistemological and ethical commitments of the action research paradigm. In doing so, it affirms action research as a credible, robust, and contextually grounded approach to generating sustainable and meaningful change.

This review suggests there is a need for an action research quality assessment tool that facilitates critical appraisal and support for four distinct activities—namely, (1) evaluation of the quality of published evidence (2) peer review, that is, to help journal editors and reviewers in manuscript appraisal and decisions (3) help researchers in preparing manuscripts and project managers in planning and conducting action research and also help readers in assessing the quality of a study and finally (4) support the identification of areas for future research improvement. The review provided an overview of the usefulness of the application of the QuARC in identifying and describing quality in action research. It also highlighted items that require more clarity in interpretation such as the essential difference between two context items on ‘previous research informing the practice concern’ and whether the project ‘has drawn on previous research’. There is also some clarity required in relation to what can be seen as an acceptable ‘level of participation’ as opposed to consultation or tokenism. It is also confusing to have an item related to whether ‘the AR project is evaluated in terms of the quality of the relationships’ included with the same wording as that of the Quality Factor of Quality of Relationships. Finally, the whole premise of quality rests in the integration of the essential factors and data related to the interlinking of the four factors, and while a critical quality concern, it was not assessed as it is not one of the QuARC items. It is recommended that, such an item be added in future iterations of QuARC along with the present scoring system. A further reflection pertains to whether the outcomes are sustainable? Assessing the sustainability of any project is likely to be beyond the limits of its published account, and might only be assessable in a follow‐up or longer term study.

### 5.6 Limitations

This research has its limitations such as the exclusion of non‐English journals and selection of action research papers from leading journals in the field of health sciences excludes action research in journals with a lower FWCI. In addition, while the exclusion of non‐self‐identified AR studies may be a limitation. However, based on our experience we deliberately wanted to include of all studies labelled by the respective authors as being action research. As a result studies were selected which subsequently transpired not be action research studies when we applied the definition of action research. Nevertheless it is also a possible limitation that the subsequent exclusion of studies that did not specify the essential elements action research as defined by the definition used in this study. While some measures of enhancing inter‐rater reliability were taken, in that all authors had a working familiarity with QuARC, nevertheless some items were found to be subject to mis‐interpretation as highlighted above. A further limitation relates to inter‐rater reliability of the scoring system and while some attempts were made to address this by each of the three raters rating the same three studies and discussing the scores. Nevertheless, caution must be exercised when interpreting the scores. Finally, the review is restricted to sources from Scopus journal articles and only focuses on the application of action research in healthcare, which may have restricted its potential in other sectors. To enhance the diversity and comprehensiveness of findings, it is necessary to broaden the database and investigate the application of AR beyond the healthcare field.

## 6 Conclusions

This hybrid narrative review addressed two research questions concerning the use of the QuARC framework to assess the quality of action research studies. In response to Research Question 1, the findings demonstrate that QuARC is a useful analytic tool for identifying how published action research studies report on quality across its 17 question items. Application of QuARC revealed substantial variation in how studies articulated quality, particularly in relation to contextual grounding, collaborative participation, and transparency in linking action–reflection cycles. While many studies demonstrated strong process rigour and clearly articulated practical outcomes, reporting on theoretical contribution, reflexive participation, and relational evaluation was less consistent.

Addressing Research Question 2, the review identified that items related to co‐researcher reflection on engagement and cycles, evaluation of relationship quality, and explicit contribution to future theory were least well addressed across the literature. These omissions suggest a tendency to prioritise action and immediate problem‐solving over reflexive theorisation and relational accountability, limiting the cumulative knowledge‐building potential of action research.

Overall, the review underscores that although the emergent and context‐sensitive nature of action research is sometimes misinterpreted as a lack of rigour, QuARC makes visible the methodological discipline inherent in high‐quality action research. Further research is needed to test the impact of the application of the QuARC tool in a systematic and theoretically underpinned manner in the methodological design to determine the usefulness of the tool as a guiding mechanism. Such research would establish QuARC as both an evaluative and developmental tool for improving the quality, transparency, and credibility of action research studies.

## Author Contributions

**Mary Casey:** conceptualization and writing – original draft. **David Coghlan:** conceptualization and writing – review and editing. **Áine Carroll:** conceptualization and writing – review and editing. **Tom Buckley:** conceptualization and writing – review and editing. **Diarmuid Stokes:** conceptualization and writing – review and editing.

## Funding

The authors have nothing to report.

## Conflicts of Interest

All authors are authors on the QuARC tool which this discussion paper is introducing. Beyond that, the authors declare that they have no known competing financial interests or personal relationships that could have appeared to influence the work reported in this paper.

## Data Availability

The data that support the findings of this study are available on request from the corresponding author. The data are not publicly available due to privacy or ethical restrictions.

## Appendix 1

Scoring system for assessing quality in action research using QuARC:

Name of Rater.

**Table:**

Factors	Questions—Please write your comments for each question item	Score as 0 (absent), 0.5 (partial), or 1 (comprehensive)
Context	Is the action driven by a practical concern?	
	Is there local, national, and international imperative influencing the practical concern?	
	Does previous research inform the practical concern?	
	Has the AR project drawn on previous research?	
		Max Mark 4
Quality of relationships	Are those who own the practical concern and have a stake its resolution included in all the steps of the action research cycle.	
	Can the participants be classified as co‐researchers?	
	Is the level of participation of the patients/clients and coresearchers evident at each step of the action research cycle?	
	Is the AR project evaluated in terms of the quality of the relationships? No	
		Max Mark 4
Quality of the action research process itself	Does the final account demonstrate a rigorous and collaborative engagement in the action research project's design?	
	Does the account demonstrate subsequent enactment of cycles of constructing, planning action, taking action and evaluating?	
	Is enactment of the cycle towards the practical and theoretical objectives transparent and reliable?	
	Is the engagement of the coresearchers evident in shared data analysis and shared reflection?	
		Max Mark 4
Outcomes	Are both forms outcomes presented (theory research) and action (practical)?	
	Are the outcomes sustainable?	
	Are the outcomes useful for other action research projects?	
	Does the theoretical outcome contribute to future theory development?	
	Is there a reflection on the process of engagement on the cycles by the co‐researchers	
		Max Mark 5
